# Perception of Law Enforcement Officers on Preventing Road Traffic Injury in Vanuatu: A Qualitative Study

**DOI:** 10.3389/fpubh.2021.759654

**Published:** 2021-12-06

**Authors:** Saen Fanai, Masoud Mohammadnezhad, Mosese Salusalu

**Affiliations:** ^1^Department of Public Health, Ministry of Health, Port Vila, Vanuatu; ^2^School of Public Health and Primary Health Care, Fiji National University, Suva, Fiji

**Keywords:** law enforcement officers, road traffic injury, risk factors, perception, Vanuatu

## Abstract

**Background:** Road Traffic Injuries (RTIs) cause approximately 1. 35 million deaths annually, and is the leading cause of death among people between ages 5 and 29. Law Enforcement Officers (LEOs) deal with Road Traffic Collisions (RTCs) and have contact with RTI victims at a daily basis, they possess an excellent perspective on preventing RTI. This study aimed to explore LEOs perceptions on risk factors and preventive measures of RTI in Vanuatu.

**Methods:** This study employed qualitative methods that used Focus Group Discussions (FGDs) to gather data from 25 LEOs between October 14th and November 30th, 2020. Self-identified Ni-Vanuatu LEOs who have been serving for over 6 months and residing at the study setting were included in this study. Purposive sampling was used to recruit study participants from three municipalities in Vanuatu. A semi-structured open ended questionnaire was designed to guide the FGDs. Data obtained were sorted out using thematic analysis processed with some preconceived themes based on theory, and also allowing the data to determine new themes.

**Results:** Data saturation was reached from conducting 5 FGDs with 25 LEOs who were traffic officers and municipal wardens. Five main themes and sixteen subthemes were generated from the study. The main themes include driving and alcohol, the challenges to effective enforcement, barriers to effective care and support for RTI victims, measures for road traffic control and promoting road traffic safety. The respondents perceived that addressing resources issues and the legislations on road traffic control act and vehicle regulation act will enhance prevention of RTI.

**Conclusion:** This study explored the risk factors of RTI and the barriers to effectively prevent RTI in Vanuatu. The study also generated suggestions of a combination of road traffic control measures that could be implemented to prevent RTI. Future research should look at effective strategies of preventing RTIs in resource deficit settings.

## Background

Road Traffic Injury (RTI) is one of the top public health concerns globally, particularly for pedestrians and passengers in Low and Middle Income Countries (LMICs) (1–3). It is the leading cause of death among people aged 5 to 29, which account for approximately 1.35 million deaths annually. Reports indicate that only 1% of the world's motor vehicles are in LMICs but 13% of deaths occur in these countries (2, 4, 5).

RTI is three times more likely to cause death in low- and middle-income countries than in high-income countries (3, 4). The number of non-communicable diseases (NCDs) and injuries has increased at an alarming rate despite improvements in the control and prevention of communicable diseases (6). People die of RTI more frequently than those who die of tuberculosis and HIV/AIDS, which are considered the world's leading causes of death (4).

By 2020, the Pacific region will witness a rise in road traffic injuries due to an increase in motorization (3). There are a variety of studies all over the world that have demonstrated the effectiveness of road traffic laws in both saving lives and preventing injuries (3, 4, 7). RTI will become one of Vanuatu's most significant causes of death and morbidity without intervention, along with diabetes and hypertension (4, 8). More than half of all road traffic accident victims are young adults and adults between 15 and 44, and they are often the primary providers in their families (9). Due to the injuries and disabilities caused by RTC, inactivity contributes to NCDs, including hypertension, diabetes, and stroke (10).

Vanuatu is one of the Pacific island countries where RTI rates are rising. Because it has not received much attention, RTI poses a public health threat (11). In high-income countries, the number of morbidity and mortality from RTI has decreased significantly, but this improvement has not occurred in low- and middle-income nations (4, 10, 12).

Few RTI studies have been conducted in the Pacific islands (11). It is necessary to expand knowledge on RTI situations in this region. Road safety will be improved by investing in preventable causes of injury (13). Due to their recurrent experiences with RTCs and their routine contact with RTI victims, LEOs possess an excellent perspective on preventing RTI (14). Most current knowledge about effective strategies for traffic law enforcement comes from extensive research on the enforcement of drink-driving and speeding based on quantitative data. But very few studies have been conducted among LEOs in this region and across the globe about their views on preventing RTI (15).

Speeding is a major cause of RTC and RTI in Vanuatu, despite a speed limit of 40 km/h in constructed areas under the Traffic Control Act (TCA) (16). According to estimates, 16 in every 100,000 people die due to traffic accidents in Vanuatu. Consequently, this number is three times greater than in Australia and Fiji, which have 5.4 and 5.8 per 100,000 respectively. Moreover, it is much higher than in the USA, where there is 10.6 per 100,000 people placing Vanuatu 91 in the world (4). Nonetheless, the data proves that there is such a plague, and that Vanuatu's roads are among the most dangerous in the region, or that its drivers are the most reckless (17).

RTI is a subject where a significant knowledge gap exists due to a paucity of study and research (9). This knowledge gap needs to be addressed urgently to ensure that the right steps are taken to prevent RTIs. Without this the vulnerable population and their families are more likely to be trapped in poverty (11). Hence, this study examines the perceptions of LEOs toward RTI prevention. The study aims to explore and understand the main causes of RTI in Vanuatu, recognize the key gaps, and recommend the best ways to improve road safety going forward.

## Materials and Methods

### Study Design and Setting

This is a qualitative study that used Focus Group Discussions (FGDs) with LEOs in three municipalities of Vanuatu between October 1^st^ and November 15^th^, 2020. In Vanuatu, these three municipalities are the only three situated in three out of six provinces, namely; Lenakel in Tafea Province, Port Vila in Shefa Province, and Luganville in Sanma Province. FGD allowed for the gathering of information much more quickly than in-dept interviews (18). The focus group process can provide a broader range of information and provide a means to clarify topics, if further clarification is needed (18–20).

### Study Sample

Participation was open to all Lenakel, Port Vila, and Luganville municipalities LEOs who meet the inclusion criteria. All male and female local LEOs who were self-identified as Ni Vanuatu, over the age of 18 years old and who had a minimum 6-month work history in any of the three municipalities were included in the study.

During the months of October and November, 2020 five FGDs were conducted to collect data. They include two FGDs in Port Vila, two FGDs in Luganville municipalities and one FGD in Lenakel municipality. Twenty five LEOs who met the study criteria were chosen purposively. To facilitate communication and also allow all participants to take part in discussions, each FGD include 5 participants (18, 19). Group discussion was continued till data was saturated and no new information is discovered in data analysis (19).

### Data Collection Tool

To provide guidance for discussions, a semi-structured open ended questionnaire was developed and it consist of seven demographic questions and five open ended questions that were usedto probe elicit information from participants and allowed participants to express their personal views freely. Participants' demographic features were collected through questions pertaining to their gender, age, education level, employment history, place of habitation, and marital status (21). To ensure that the questionnaires were suitable to the respondent needs, pre-tests were conducted to ensure the questions, instructions, and language used were clear. Using the results from the pre-test, flaws in the questions were corrected, and re-adjustments were made to meet response expectations.

### Study Procedure

Before reaching out to potential participants, formal communication with respective LEO authorities were established. Prior to the scheduled FGDs, information sheets in English and Bislama (Vanuatu's common language) were handed out to potential study participants. Participants had to complete and sign a consent form in order to participate in this study. Witnessed verbal consent and permission to audio-record the discussions were obtained. Participants were assured of confidentiality, allowing them to provide information freely. To ensure there would be no influence from other potential study participants, the main researcher conducted the FGDs in isolation. The FGDs were conducted in a way whereby simple, general questions were asked, thus gradually progressing to more specific questions. For the FGD, participants were asked to fill out their demographic information according to the questions asked, and then return the papers to the researcher. Throughout the FGDs probing techniques were performed according to the reflections of each participant, concerning experiences and their perceptions of RTIs. After each FGDs, preliminary data analysis took place simultaneously in order to identify ideas that can guide the next FGDs. The main researcher conducted all the FGDs in Bislama. To avoid exhaustion, which, in turn, would negatively affect the quality of questions asked, the principal researcher conducted only one FGD a day to ensure convenience for all participants during the course of the study. Depending on the responses, each interview took 45 to 60 min. All FGDs were recorded using a voice recorder. In the process of collecting data, the researcher accumulated data on every concept until saturation was reached and further collection was unable to provide any new information (22).

### Data Management and Analysis

Microsoft Word was used to transcribe data from digital recorders and notes taken during FGDs. A researcher and research assistant reviewed each FGD transcript twice-immediately following transcription and again during data analysis. A comparison was made between the transcripts and digital recordings for accuracy. Discussions and triangulation of data sources resolved any disagreements or issues which needed clarification. Manual thematic analysis was performed for these FGDs based on exploring both predetermined issues of interest and looking for new issues raised by the respondents (23, 24). In order to adequately represent the topic area, the themes were listed and coded based on frequency and order of mentions. Open coding was done and codes were grouped into categories, and themes identified. Data analysis involved summarizing large raw data quantities, categorizing and rearranging of the data. To facilitate further analysis, the collected data was sorted and tabulated in a form of tables (25).

### Study Rigor

Credibility, confirmability, transferability and reliability were ensured to guarantee rigor. Credibility was enhanced by researcher's field experiences, their engagement with participants, data collection and analysis, and data triangulation that included FGD and peer review. In order to achieve a high level of confirmation, the first Author as the principal investigator implemented triangulation and peer-reviewing strategies. For transferability, the researcher explained how the data was collected and analyzed, and how the results were interpreted. Finally, the study outcome can be relied upon because the development process was described sufficiently in detail to facilitate a replication by another researcher.

### Ethical Considerations

Ethical approval was obtained from both the Vanuatu Ministry of Health Research and Ethics committee and the College Health Research & Ethics Committee (CHREC) of the Fiji National University (FNU). All potential participants were provided with information explaining the purpose of the study, both verbally and in writing. Written consent was mandatory and collected from all participants.

## Results

### Demographic Characteristics

The demographic characteristics of the respondents are summarized in Table 1 below. Twenty-five officers including police officers and municipal wardens who are mainly involved in traffic law enforcement participated in this study and they all agreed to participate. The majority of the total respondents were male (72%) but almost equally distributed by age, the majority (52%) ages and above 41. According to their level of education, the majority (60%) completed high school level. The length of time the respondents were employed was almost equally distributed by tertiles, with more than a third (36%) employed between 11 and 20 years. The majority of these participants (52%) grew up in town. And according to their marital status record, eighty percent (80%) of them are married at the time of this study.

**Table 1:** Profile of law enforcement officer respondents (*n* = 25).

**Respondents**	**Categories**	**Frequency (*n*)**	**Percentage (%)**
Gender	Male	18	72
	Female	7	28
Age	25–40 years old	12	48
	41–60 years old	13	52
Level of Education	≥Tertiary	10	40
	High School	15	60
	Primary School	0	0
	No school	0	0
Number of years	0–10 years	8	32
employed	11–20 years	9	36
	≥21 years	8	32
Grew up	In town	13	52
	In the Village	12	48
Marital status	Married	20	80
	Single	5	20

### Perceptions of LEOs

Based on the FGDs, the following main themes emerged; driving and alcohol, challenges to effective enforcement, barriers to effective care and support for RTI victims, measures for road traffic control and promoting road traffic safety. Seventeen sub-themes arose from these five main themes (Table 2). Further thematic analysis of LEOs FGDs are described below under each main themes and sub themes. The sub themes are supported with samples of interview responses that are quoted. Each interview sample quoted is labeled with the age and gender of the respondent including the unique participant identification number LEO1 representing law enforcement officer participant 1 and so on.

**Table 2:** Main themes, sub-themes and codes for LEO.

**Theme**	**Sub-theme**
Driving and Alcohol	Lack of public responsibility
	Permissive and accepted
	Entertainment
Challenges for effective	Work overload
enforcement	Lack of resources
	Ineffective traffic law
Barriers to effective care and	Lack of first aid knowledge
support for RTI victims	Lack of first aid equipment
	Weak medical emergency
	communication system
Road traffic control measures	Road infrastructures
	Vehicle road worthiness standards
	Road traffic act
Promoting road traffic safety	Stricter penalties
	Non-monetary penalties
	Traffic law enforcement
	Awareness intervention

### Theme 1: Driving and Alcohol

Three subthemes that included lack of public responsibility, permissive or accepted and being a source of entertainment emerged when asked about the participant's views on the common causes of RTI. They described alcohol among other causes as strongly associated with RTCs and injuries.

#### Lack of Public Responsibility

While individuals often have the ability to take care of their own health, they lack the ability to protect or promote health at the population level. When it comes to drinking alcohol and driving the respondents blame the public for not taking responsibility to report to the police or other authorities. One of the respondents stated,

“*Vehicles are often spotted with drunk drivers but no one cares to report it, we are trying to copy the foreign ideology to “mind your own business” and it is not helping our communities.”* (FGD 46 years old male, LEO3)

People over rely on police to do their job, another respondent stated,

“*The general public take it for granted that it is the responsibility of the police to find drunk drivers and arrest them but police cannot be everywhere at one time.”* (FGD 33 years old male, LEO11)

There is need for community support to the work of police, one respondent added,

“*If our communities join the police take some responsibility to tackle the issue of drinking and driving, our roads and the communities will be safe.”* (FGD 49 years old male, LEO13)

#### Permissive and Accepted

Drinking and driving appear to be permitted and has become an acceptable culture in most communities. One of the respondents stated,

“*Many passengers are okay with drivers who had a couple of kava shells or who have had alcohol but is able to drive.”* (FGD 29 years old male, LEO20)

Drunk drivers know they won't be reported, another respondent added,

“*As it appears to be acceptable by the community, people continue to drink and drive knowing they won't be reported by the community if the police do not catch them.”* (FGD 39 years old male, LEO21)

The issue has become too common, one respondent stressed,

“*Because it happens all the time, no one cares to report to the police when they notice drunk people driving.”* (FGD 33 years old female, LEO6)

#### Entertaining

The experience of drinking and driving has come to be strongly associated with amusement, so that one common understanding of the idea is for fun and laughter, although many times the outcome is devastation. The respondents view that many young drivers like to drink and drive,

“*People find drinking and driving enjoyable that is why a lot of accidents are related to drinking and driving.”* (FGD 48 years old female, LEO17)

Drinking and driving is a form of celebration entertainment, one respondent added,

“*Drinking and driving appear to be traditionally accepted so drivers use it as a form of celebration and entertainment for themselves and their peers.”* (FGD 45 years old female, LEO16)

In addition, young people in particular find riding in a vehicle with drunk drivers entertaining. One of the respondents stated,

“*It is dangerous but many young people enjoy being in the vehicle with drunk drivers because of free riding, loud music and just showing off.”* (FGD 39 years old male, LEO21)

### Theme 2: Challenges to Effective Traffic Law Enforcement

Three subthemes including workload, lack of resources and ineffective traffic law were generated when asked about the challenges faced with enforcing traffic laws. The respondents claim that unlike other crimes, trafficking is not a single and static event. It involves multiple offenders and crime sites making enforcement challenging.

#### Workload

Work overload takes a heavy toll on law enforcement officers. Having little control over an overwhelming workload can lead to burnout. The respondents think they are overwhelmed and exhausted with their responsibilities thus cannot implement enforcement to the level expected by everyone.

One of the respondent stated,

“*Officers are constantly under pressure due to other policing workload with little resting time thus we cannot offer the traffic law enforcement at a level expected of us.”* (FGD 47 years old male, LEO12)

Police do not really get the rest they need, one respondent stated,

“*Even during our resting time at home we work over phone if not recall for duty when the situation requires, so being everywhere on the roads most of time as expected by the people is practically impossible.”* (FGD 49 years old male, LEO13)

Leadership roles are easily distracted, one respondent added,

“*Our senior officers often get sick because of stress and develop mood disorders that subsequently affect their leadership roles, all this relating to the level of workload we are faced with at a daily basis.”* (FGD 32 years old female, LEO15)

#### Lack of Resources

The underlying cause of a number of challenges discussed were the lack of resources. According to the respondents, to improve the enforcement of traffic laws sufficient human and appropriate material resources are required. One of the respondent stated,

“*The number of vehicles on the roads are increasing by hundreds every day but police recruitments happen only every 5 to 10 years, this will not solve our human resource issues consequently the enforcement of laws including traffic laws.”* (FGD 45 years old female, LEO16)

From the discussions, it was also noted that given the low number of officers under different jurisdictions, officer retention per unit is impossible and experienced traffic officers had to be rotated. The respondents stated,

“*We always have new traffic officers with countable senior and experienced officers in the traffic unit as due to short staff, staff rotation cannot allow experienced traffic enforcement officers to remain in the traffic unit.”* (FGD 35 years old male, LEO5)

Material resources including traffic enforcement vehicles are inadequately distributed making enforcement difficult. One respondent stated,

“*Without traffic vehicles we can do very little to effectively enforce traffic laws. Traffic law enforcement requires a lot of mobilization, if we could have at least motorbikes it would have help.”* (FGD 45 years old male, LEO9)

#### Ineffective Traffic Law

According to the respondents, their general understanding of traffic laws is that it is there to make our roads and driving safer but if enforced and the outcome proofs otherwise then it shows that the laws are not working. The respondents who are quiet familiar with the traffic laws perceived that the traffic law is no longer effective and need amendment in various sections. One of the respondent stated,

“*It is time our traffic laws are reviewed and unless this are done, our RTC situation will not improve.”* (FGD 42 years old male, LEO23)

The traffic control act is becoming unsupportive, one other respondent stated,

“*Our traffic law is old, it was last amended more than fifteen to twenty years ago, our road conditions cannot support the current traffic load therefore we must review and amend our traffic laws.”* (FGD 49 years old male, LEO13)

The traffic control act determines new development for traffic control, one respondent stated,

“*If we are to introduce traffic lights, Breathalyzer and others that contribute to road safety our traffic law have to support the initiative, currently we can't.”* (FGD 44 years old male, LEO22)

The traffic control act also determines the Penalties for traffic law offenders, another respondent stated,

“*Penalties for traffic law offenders appear to be too weak; this can only be change with our traffic laws amended.”* (FGD 49 years old male, LEO13)

### Theme 3: Barriers to Effective Care and Support for RTI Victims

Three subthemes that include the lack of first aid knowledge, the lack of first aid equipment and the weak medical emergency communication system emerged when asked about what law enforcement officers could do to improve care of RTI victims. The respondents claim that addressing these key barriers can allow for effective care of RTI victims.

#### Lack of First Aid Knowledge

The respondents perceived that the lack of first aid knowledge results in ineffective care of RTI victims. One respondent stated,

“*It is necessary to conduct first-aid training for police officers as most of them do not apply appropriate first aid care to RTI victims.”* (FGD 38 years old male, LEO12)

LEOs require relevant trainings for specific injuries, another respondent added,

“*It is also essential that officers attend workshop on care for different types of injured patients as officers are not comfortable to deal with all injuries.”* (FGD 43 years old male, LEO8)

#### Lack of First Aid Equipment

The respondent believed that if appropriate first aid equipment is made available to all emergency respondents it could make a difference to the outcome of care. One of the respondent stated,

“*With the already lack of resources to enforce traffic laws, it is even worse to deal with traffic injuries. There are no first-aid kits and stretchers.”* (FGD 45 years old female, LEO16)

With lack of appropriate equipment unsafe practices are engaged, another respondent stated,

“*We only use cloths or plastic bags to prevent blood contact but most of the time we care for victim with bare hands.”* (FGD 48 years old female, LEO17)

Private vehicles are utilizing in the absence of ambulances, one respondent added,

“*In most occasion there is no ambulances at scene, we had to transport victims in private cars. The police should have fully equipped vehicle to provide appropriate care for RTI victims.”* (FGD 38 years old female, LEO19)

#### Weak Medical Emergency Communication System

The respondents feel there is need to improve the medical emergency communication system that effectively respond to RTI emergencies. They perceived that the weak medical emergency communication system cause delay to effective care for RTI victims. One of the respondent stated,

“*It is necessary to have available standby ambulance along main roads and they must have special and free mobile phone numbers to be reached.”* (FGD 51 years old female, LEO25)

Communication could be improve using free lines, another respondent added,

“*The hospital emergency line is unreachable. All the hospitals must have a unique free emergency number to improve communication.”* (FGD 39 years old male, LEO21)

One respondent comment on poor collaboration because of proper communication,

“*There are often communication issues with other emergency providers, confusions cause unnecessary delays to deliver care. All emergency care provider must develop a communication network system that allows for easy access.”* (FGD 42 years old male, LEO23)

To ease communication issues, one respondents stressed,

“*All emergency numbers should be free and easy to remember.”* (FGD 51 years old female, LEO25)

### Theme 4: Road Traffic Control Measures

Three subthemes including road infrastructures, vehicle road worthiness standards and the road traffic act emerged when discussing the prevention of RTC and injuries. The respondent's state specific road control measures that are deemed necessary to address RTI situation in the country.

#### Road Infrastructures

According to the respondents, infrastructure improvement reduces RTC, RTIs, and fatalities when it promotes safe roads for pedestrians, vehicles and cycling. The respondents discussed specific road infrastructures that they perceived would improve road safety including reconstructions of roads and footpaths. One of the respondent stated,

“*The rapid increase of vehicle volume required urgent need for more roads or widening of the current roads.”* (FGD 43 years old male, LEO8)

One respondent comment on the need for footpaths,

“*There is need for proper footpaths in high traffic roads.”* (FGD 45 years old female, LEO16)

Furthermore, the issue of road traffic signs was discussed. Traffic controls include traffic signals, traffic signs and pavement markings should be erected. One respondent stressed,

“*It is about time Vanuatu starts using traffic lights, the traffic flow is out of control.”* (FGD 47 years old male, LEO10)

In the absence of road signs the roads are not safe, one respondent stated,

“*Our roads do not have traffic signs and this is frightening. Damage traffic signs need to be replace, new traffic signs put up and pavement markings must be done.”* (FGD 33 years old female, LEO6)

#### Vehicle Road Worthiness Standards

The respondents think that roadworthiness of a vehicle contributes to road safety. They agreed that all vehicles on the road must be in suitable condition to operate and in line with technical standards required under the traffic law. The respondents stated,

“*Some vehicles do not appear safe but they are on the road and still have roadworthiness certificate.”* (FGD 29 years old male, LEO2)

There is concern on the safety standards of vehicle imported, one of the respondent stated,

“*While importing and selling used vehicle, the main concern is to check whether or not the vehicle imported matches up with the safety standards of the country.”* (FGD 44 years old male, LEO1)

The discussions mention authorities that are responsible to issue roadworthiness certificates, they stated,

“*It is important to inspect all the vehicles properly as fully maintained and safe vehicle is less likely to involve in a road accident.”* (FGD 41 years old female, LEO18)

#### Road Traffic Act

The respondents perceived that our road should be safe when existing traffic laws are enforced and that everyone can trust that all road users comply with traffic rules. According to the respondents, the current road traffic Act that regulates road traffic, the use of vehicles and the user of roads including other purposes is outdated. They stated,

“*There is need to review the current road traffic act, what we have is very old and was drafted when we had few roads with fewer vehicles on the road.”* (FGD 49 years old male, LEO13)

The respondents perceived that the old traffic law make it difficult to enforce road safety measures in the current stage. Generally, the respondent stated,

“*The current traffic act limits our ability to prevent unsafe vehicle driver or passenger practices.”* (FGD 38 years old male, LEO19)

As far as new road safety measures are concerned, the respondents also expressed that the current traffic laws do not gather for the introduction of new safety road measures,

“*We cannot use Breathalyzer to identify and penalize drivers who are under the influence of alcohol because our traffic laws do not gather for the use of Breathalyzer, we need to review the traffic act urgently.”* (FGD 42 years old male, LEO23)

### Theme 5: Promoting Road Traffic Safety

Four subthemes that include stricter penalties, non-monetary penalties, enforcement and awareness interventions were generated when asked about policy solutions and way forwards to promote road traffic safety.

#### Stricter Penalties

According to the respondents, making speeding, alcohol-impaired driving and reckless driving a serious criminal offense can significantly reduce road traffic injuries and deaths. They perceived that the current penalties for traffic offenders are not severe enough thus people often reoffend. The respondents stated,

“*Traffic law offenders should go to jail, monetary penalties are cheap and people do not learn lessons by being able to pay their way out.”* (FGD 49 years old male, LEO13)

Increase fines is a form of stricter penalties one respondent stated,

“*We should increase fines to a level that the average drivers are unable to pay so that they go to jail when to they fail to pay.”* (FGD 29 years old male, LEO14)

One respondents added the current penalty is too small and is not helping,

“*Currently, traffic offenders are only charged 5,000VT (equivalent to USD$35) when booked. Weak penalties like this cannot help the situation.”* (FGD 43 years old male, LEO8)

#### Non-monetary Penalties

The respondents also agree that while an increase of fines for traffic offenders could in a way reduce RTC it was also discussed and suggested that non-monetary penalties like community services be trial as it is known to have worked for other countries. The respondents stated,

“*In other countries they are using community services for traffic law offenders and it is working for them, we should explore this and give it a try.”* (FGD 45 years old female, LEO16)

Community services as an alternative, some respondents also stated,

“*We should do away with monetary penalties because it is becoming affordable, all road traffic law offenders should be jailed or serve community services,”* (FGD 37 years old male, LEO7)

One respondent stated in addition to,

“*Cease driving license for traffic law offenders for a period of 5 years.”* (FGD 44 years old male, LEO22)

#### Enforcement

Noted from the discussions, the physical presence of police officers in high traffic areas is necessary for road safety. The respondents stated,

“*People are demanding physical presence of police officers in traffic risky areas, it does have an impact. Drivers appear to be sober and in good manners when police officers are around. You hardly see careless driving or driver under influence of alcohol when there is presence of police on the road.”* (FGD 48 years old male, LEO4)

The respondents acknowledge that when traffic enforcement officers are not around RTCs happen. One respondent stated,

“*It is common that when traffic law enforcement is weak or unavailable in certain areas accidents occur.”* (FGD 49 years old male, LEO13)

#### Awareness Intervention

According to the respondents, road safety campaigns are necessary means of influencing the public to behave more safely in traffic. It was clear from the discussion that there is need for regular road safety awareness on radio, TVs and posters in public places. One of the respondent stated,

“*There is very few awareness to promote road safety, there is a need and we should be making more public awareness through radio or TV.”* (FGD 35 years old male, LEO5)

Another respondent expressed,

“*It is the responsibility of Police and health to come up with brochures and posters and distribute to the general public to promote road safety.”* (FGD 46 years old male, LEO3)

According to some respondent role modeling is a powerful teaching tool for passing on the knowledge, skills, and values of safe driving. They stressed,

“*We should be leading by example. If we are to make a difference, our conduct and practice in public places must be seen as law abiding. We have law enforcement members who are traffic law offenders themselves; this is not good role modeling.”* (FGD 38 years old female, LEO19)

## Discussion

This study explored the perception of LEOs on preventing road traffic injuries in Vanuatu. The findings were summarized under the themes the challenges to effective enforcement, barriers to effective care and support for RTI victims, measures for road traffic control and promoting road traffic safety.

### The Challenges to Effective Enforcement

The key challenges highlighted in this study include inadequacies in the road control traffic act and the vehicle regulation act, and the lack of resources.

The findings in this study indicate that the current road control traffic act and the vehicle regulation act is inadequate therefore a major barrier to road traffic safety developments. The findings suggest that the current road and traffic legislations does not allow for road safety control initiatives such as the introduction of traffic lights, the use breathalyzers as evidence to prosecute people who drink alcohol and drive, and the lenient penalties for traffic law offenders making enforcement ineffective. The findings indicate that unless the road traffic control act is reviewed, new traffic control measures aimed at preventing RTI may never be realized. The lack of strengthened traffic legislation is a tendency for people to expect extremely lenient treatment from the traffic police, a low level of fines for traffic violations and a lack of long-term sustainable planning are important obstacles. These situations can lead to a lack of effective enforcement (13). Furthermore, vehicle regulation represents an important step toward safer vehicles, but certainly offers no guarantee that vehicles are used as intended and that they will be properly maintained (26). It is however noted in various study findings that vehicle regulations represent an important road safety issue for developing countries (27). Countries should be making vehicles more protective for occupants, pedestrians and cyclists; and formulating and implementing transport policies that encourage safety (27, 28). Moreover, several countries have achieved reductions in the figure of crashes and injuries by creating and implementing stricter traffic laws, including better policing of alcohol use on the road; speed limits; the use of seat-belts and child restraints; crash helmets (29). Legislation in itself such is not a safety measure. A safety effect demands more. Before introducing legislation, it is recommended to foster a positive attitude amongst the general population and amongst social groups via publicity campaigns (26). Once the new law becomes operational, it is necessary to inform road users about the content and intent of that law. The public should also be informed about the possible consequences of breaking the law (29). Also, the road user must be aware that the legislator is serious about application of the law. This can be demonstrated by ensuring that police apply sufficient effort to enforce the law and that the judiciary can take care of any criminal procedures.

The findings marque the lack of resources characterized by lack of human resources and equipment as the root cause of ineffectiveness to prevent RTIs. The findings suggest that the lack of road safety education programs, weak road traffic law enforcement and the lack of physical presence of law enforcement officers on the roads are indications of the challenges faced with human resources and equipment including vehicles. However, according to Quansah R et al. (30) when looking at the availability of equipment noted that the lack of planning, rather than resource restrictions, were the main reasons for the absence of such vital resources that include equipment. Their findings suggest that improved administration could also strengthen utilization of resources. A frequent problem is that experience in road safety is given less priority in career advancement than is criminal investigation. Members of the police who receive extra training in road safety often spend a limited time in road safety units before being transferred. Efforts to increase retention of those with skills acquired in road-safety units are needed (31). Nevertheless, it could also be added that the lack of good coordination to maximize use of the scares resources makes the situation even worse. According to WHO, prevention of RTIs is a shared responsibility and needs multi-sectoral collaboration (1, 8). Collaboration might take the form of research, information sharing, policy development, advocacy and capacity development (27, 32). Lack of coordination is one important barrier, which was in line with a study in the case of post-crash events (33).

### Barriers to Effective Care and Support for RTI Victims

The results in this study indicate that capacity building for LEOs on first aid with specific focus on different types of injuries can be life saving for RTI victims. The findings indicate that many police officers and municipal wardens who most frequently a first responders to RTCs have no idea how to attend to RTIs and in most cases they can only secure the sites and wait for hospital emergency response team or pro-medicals to arrive and attend to the injuries. Huge delays and failure to provide appropriate on the spot actions have cost many lives. This finding is consistent with the findings of Larsson EM et al. (34) in their study on first-aid training and bystander actions at traffic crashes. Intensified first-aid training of the general public could lead to citizens who are more cautious in traffic and to bystanders who provide more immediate and adequate first aid at traffic crashes and other emergencies. Other studies reveal that most crash scene care providers perform no intervention at all, or even impede the provision of proper care due to limited knowledge and skills (35). A case presentation in Iran highlights that over-crowding and laypeople interference at the scene disrupts the relief and rescue of RTI victims (36). Working with limited knowledge during lifesaving time not only decreases chances of survival for injured victims but also increases the risk of a care provider becoming a victim immediately or in the near future (37). Furthermore, according to Chokotho L et al. (38) mandating first aid training for drivers, especially for the commercial drivers who account for a sizeable proportion of all road miles driven may be particularly effective because other drivers are often the first people not involved in a crash to encounter the victims. Several studies have shown that even short training sessions for drivers can improve responses. Generally the major barriers for preventing RTI are human factors (13).

### Measures for Road Traffic Control and Promoting Road Traffic Safety

The findings suggest that community education on road traffic issues and enforcement of road traffic control laws as measures to prevent RTI in Vanuatu. The respondents claim that road users including vehicle drivers, passengers and pedestrians have inadequate knowledge required for road safety. Moreover, they perceived that the general population lack the information on RTI situation and how to prevent RTI. The lack of awareness on TV, Radio and newspapers is evident to the lack of information on the issue. The findings therefore suggest that community and school education programs on road safety is fundamental for preventing RTI. This is consistent with a study conducted by Olivier D et al. on safety education of pedestrians for injury prevention. They find that pedestrian safety education can change observed road crossing behavior (39). Campaigns combined with increased police enforcement appear to be more effective than campaigns without (40). However, in contrast, a study conducted in Kenya by Odhiambo W et al. (41) on the impact of road safety awareness campaign on motor cycle related road traffic injuries showed that the compliance with road safety measures among motor cycle passengers remain low after road safety after mass awareness campaign intervention. Further inconsistency was observed in another study, their review indicates that there is no reliable evidence supporting the effectiveness of pedestrian education for preventing injuries in children and inconsistent evidence that it might improve their behavior, attitudes, and knowledge (42). Further review of similar literature is necessary to draw further conclusions. Another common perception on preventing RTI in Vanuatu is traffic law enforcement. The findings suggest that effective road traffic law enforcement will improve prevention of RTI in Vanuatu. The inefficiency of rolling out traffic law enforcement activities does not mean that law enforcement does not work for Vanuatu. Law enforcement can be improved by addressing key barriers for effective interventions. This finding is contrary to a study conducted in Botswana by Mphela T et al. (7) who their findings reveal that the enforcement of the new road laws has achieved little in the reduction of fatalities. Increasing the minimum driver licensing age may be a panacea to road accidents. Licensed drivers in the age group 30 to 45 years have the lowest rate of fatalities (7). Other studies indicate that the lack of strengthened traffic legislation, a tendency for people to expect extremely lenient treatment from the traffic police, a low level of fines for traffic violations and a lack of long-term sustainable planning are important obstacles (13). These situations can lead a lack of effective enforcement. The experience from high-income countries shows that if road-users had greater respect for road traffic legislation, the number of road crash fatalities could be almost halved (40).

### Strengths and Limitations

Researchers in the study gathered the views of traffic LEOs on preventing RTC in Vanuatu, a resource-stricken nation with rapid motorization and RTIs. This is one of the few studies conducted in this region focusing on ways to improve the current situation using a qualitative approach. Although, this study applied a rigorous design that contributed to the trustworthiness of the results, it had few limitations. The number of participants was relatively small and this study only focused on LEOs in three municipalities of Vanuatu.

## Conclusion

This study explored the risk factors of RTI and the barriers to effectively prevent RTI in Vanuatu. The study also generated suggestions of a combination of road traffic control measures that could be implemented to prevent RTI. Future research should look at effective strategies of preventing RTIs in resource deficit settings. Furthermore, specific road safety control measures require more assessment and evaluation to better understand its effectiveness in LMIC countries like Vanuatu.

## Data Availability Statement

The original contributions presented in the study are included in the article/supplementary material, further inquiries can be directed to the corresponding authors.

## Ethics Statement

The studies involving human participants were reviewed and approved by College Health Research and Ethics Committee (CHREC) of the Fiji National University (FNU). The patients/participants provided their written informed consent to participate in this study.

## Author Contributions

SF developed proposal, collected data, and analysis data. All authors have contributed to the concept, design, writing and revising of the manuscript, and have also approved for the submission.

## Funding

This study was funded by the Vanuatu Ministry of Education's Training and Scholarship Unit.

## Conflict of Interest

The authors declare that the research was conducted in the absence of any commercial or financial relationships that could be construed as a potential conflict of interest.

## Publisher's Note

All claims expressed in this article are solely those of the authors and do not necessarily represent those of their affiliated organizations, or those of the publisher, the editors and the reviewers. Any product that may be evaluated in this article, or claim that may be made by its manufacturer, is not guaranteed or endorsed by the publisher.
